# North Atlantic minke whale (*Balaenoptera acutorostrata*) feeding habits and migrations evaluated by stable isotope analysis of baleen

**DOI:** 10.1002/ece3.8224

**Published:** 2021-10-24

**Authors:** Dafne Eerkes‐Medrano, David C. Aldridge, Arnoldus S. Blix

**Affiliations:** ^1^ Aquatic Ecology Group, Department of Zoology University of Cambridge Cambridge UK; ^2^ St. Catharine's College Cambridge UK; ^3^ Department of Arctic and Marine Biology UiT‐The Arctic University of Norway Tromsø Norway; ^4^ Present address: Marine Laboratory Marine Scotland Science Aberdeen UK

**Keywords:** baleen, diet, migration, minke whale, stable isotopes

## Abstract

Isotopic analyses of the incrementally growing baleen in Mysticeti have been used to learn about their feeding and movement patterns. Using methods previously applied to Pacific minke whales, stable δ^15^N and δ^13^C isotope values were measured along the baleen plates of male and female minke whales from two locations in the Northeast Atlantic. The sample sizes used in this study are comparable to those previously used in the literature, and, although limited in size, the evidence suggests differences in isotopic signatures between whales caught at different locations. Both the δ^15^N and δ^13^C data suggest whales at the higher latitude site of Svalbard have a narrower diet than the whales from Lofoten/Vesterålen in Norway. Across all whales, the δ^15^N data indicate the whales primarily prey on fish for much of the year, only switching to zooplankton during the spring bloom. The δ^13^C data fail to confirm whether the whales migrate over long distances.

## Graphical abstract

Stable isotopes were measured in the baleen plates of minke whales to investigate seasonal patterns in diet. Whales prey on fish for most of the year but switch to zooplankton during the spring bloom. We propose that not all minke whales migrate, as previously assumed, and this could have important implications for understanding food webs in the Norwegian Sea. (Blue dots are δ_15_N isotopes, and red triangles δ_13_C isotopes).

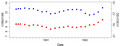

## 1 INTRODUCTION

Minke whales are important constituents of the Northeast Atlantic ecosystem as predators and as a species harvested commercially in Norway and Iceland. Much emphasis has been placed on abundance estimation (Skaug et al., 2004) and studies of population genetic structure (Quintela et al., 2014). In terms of their trophic interactions and food‐web roles, Nordøy and Blix (1992), Haug et al. (1996) and Windsland et al. (2007) have described minke whale stomach contents in summer, but stomach contents can only provide snapshots of what the whales eat in a specific area. As their behaviour is elusive and their size is small, consequently there is much that remains unknown regarding their behaviour, seasonal feeding habits, migrations, and winter distributions.

Large migrations and feeding activity across extensive areas are characteristics of a variety of mysticete whales. Given the ethical considerations of obtaining sufficiently large sample sizes to study whale feeding and movement habits, either non‐destructive methods are employed, such as telemetry (Lee et al., 2017; Milmann et al., 2020) or biopsy darts that collect skin and blubber (Witteveen et al., 2009); or smaller samples are taken when opportunities allow, such as through strandings and museum samples (e.g. Best & Schell, 1996), licensed hunts (e.g. Hobson & Shell, 1998), or active research programmes (e.g. Mitani et al., 2006). Among the materials sampled, whale baleen is a keratinised structure that grows continuously. Application of stable isotopic analysis (SIA) to baleen plates can provide long records of whale feeding and movement habits. This method has been applied in a variety of whales, such as southern right whales (*Eubalaena australis*) (Best & Schell, 1996); bowhead whales (*Balaena mysticetus*) (Hobson & Shell, 1998; Schell et al., 1989); Pacific minke whales (*Balaenoptera acutorostrata*) (Mitani et al., 2006); and Antarctic minke whales (*Balaenoptera bonaerensis*) (Uchida et al., 2021). Findings from these studies have provided insights into migratory habits (Best & Schell, 1996; Schell et al., 1989) and seasonal feeding patterns and prey preferences (Mitani et al., 2006; Uchida et al., 2021).

The stable isotope analysis of Pacific minke whales provided insights into feeding shifts from krill to fish, but were unable to resolve latitudinal migrations (Mitani et al., 2006). In the case of the North Atlantic minke whale, while descriptions of their stomach contents in summer have been recorded (Haug et al., 1996; Nordøy & Blix, 1992; Windsland et al., 2007), a greater understanding of their year‐round feeding habits is still needed. With regards to their movements, outside of the summer season little is known. It is assumed that they spend the summer at high latitudes and migrate to temperate or even tropical waters to breed and give birth in winter (Jonsgård, 1951; Risch et al., 2014; Vikingsson & Heide‐Jørgensen, 2015), but others have questioned whether there may be an alternative to all individuals undertaking seasonal migrations to lower latitudes (Corkeron & Connor, 1999; Jonsgård, 1966; Kavanagh et al., 2018).

**FIGURE 1:**
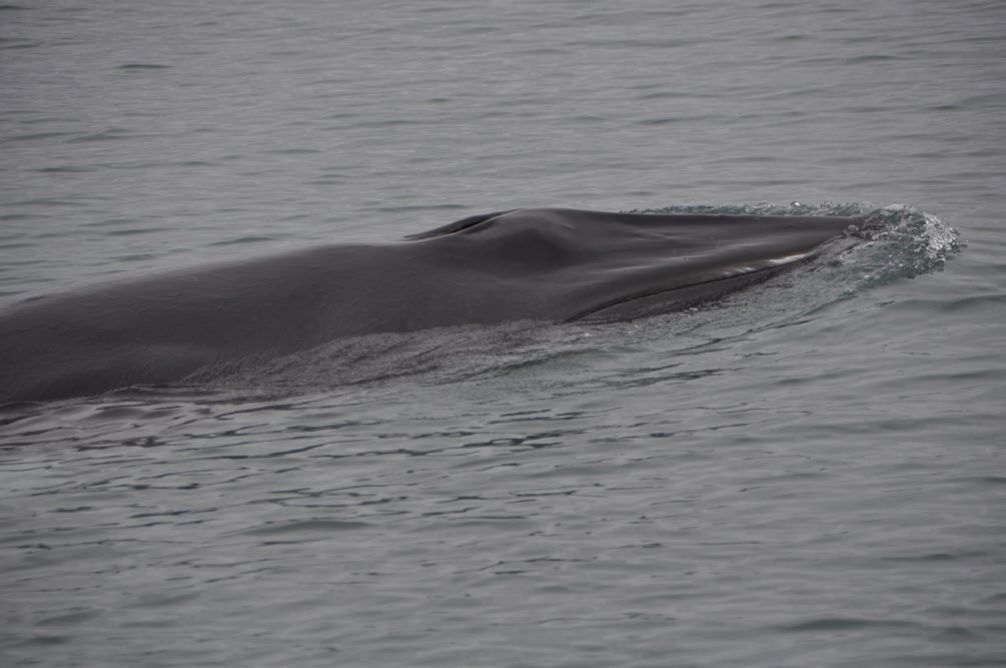
A minke whale *Balaenoptera acutorostrata* surfacing north of Svalbard (photo: Arnoldus Schytte Blix)

This study applies stable isotope analysis to understand feeding and movement patterns of minke whales caught along the coast of Northern Norway and Svalbard. The same methods used for Pacific (Mitani et al., 2006) and Antarctic (Uchida et al., 2021) minke whales are applied in the present study. Patterns in δ^15^N and δ^13^C are presented with an interpretation of what these reflect about the whale feeding habits. Also size measurements and estimated growth rates for the baleen plates are presented. In light of advancements to existing technology, the availability of new technologies and the increasing application of inter‐disciplinary research, this study also takes the opportunity to collate existing literature on North Atlantic minke whale biology, behaviour and sightings to support an alternative hypothesis to the north–south migration assumed of these animals (Figure 1).

## 2 MATERIALS AND METHODS

### 2.1 Animals

**FIGURE 2:**
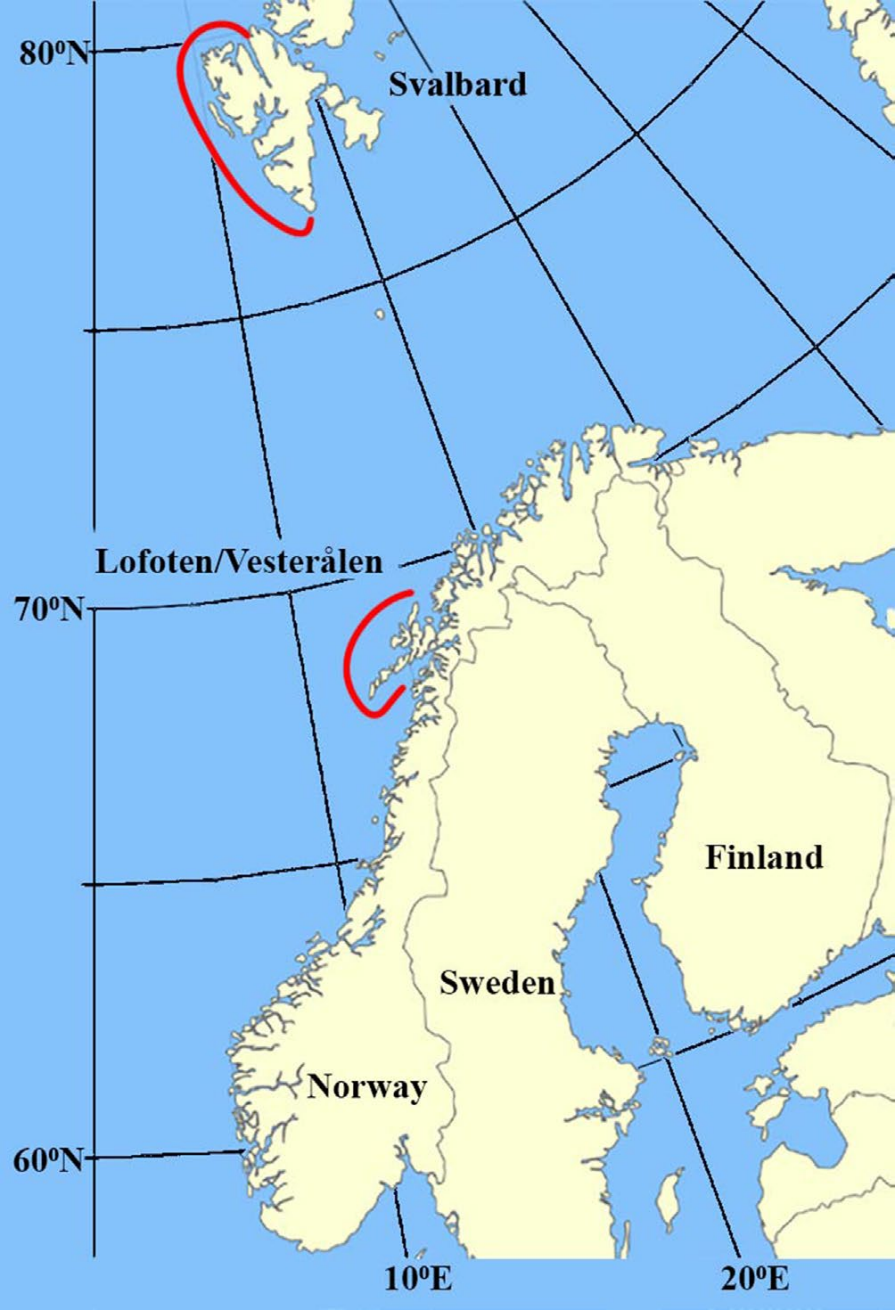
Map showing the areas (red line) within which the minke whales were harvested off Lofoten/Vesterålen and Svalbard

A total of 15 minke whales (*Balaenoptera acutorostrata*) were examined (Table 1). These were taken under scientific permit from the Norwegian Ministry of Fisheries, as part of the Norwegian Scientific Whaling Program (1988–1994), in various locations in Lofoten/Vesterålen (L/V) (67°30″–69°00″N; 12°00″–15°30″E) and in Svalbard (S) (76°00″–79°30″N; 09°00″–18°00″E) (Figure 2) in July and August of 1992.

**TABLE 1:** Lofoten/Vesterålen (L/V) captured *male* (LV2, 5, 8, 9, 12) and *female* (LV1, 7, 10, 15) and Svalbard (S) captured *female* (S1, 4, 7, 9, 10, 11) minke whales, indicating whale ID; sex and whether pregnant; length of whale (cm); length of baleen plate (cm) (i.e. maximum length exclusive of free bristles; Jonsgård, 1951); length of baleen plate and bristles; distance measured between the δ^15^N dips (cm); date of capture; and estimated dates of minimum δ^15^N

ID	Male(M) or Female(F) (‘p’ if pregnant)	Length of whale (cm)	Length of baleen plate, excluding bristles (cm)	Length of baleen and bristles (cm)	Distance between δ^15^N dips (cm)	Date of capture (1992)	Estimated date of first δ^15^N dip	Estimated date of second δ^15^N dip
LV2	M	745	18.1	26.9	13	06‐Jul	07‐Jun‐92	08‐Jun‐91
LV5	M	712	16.4	24.6	12	21‐Jul	29‐Jun‐92	30‐Jun‐91
LV8	M	824	19.7	31.2	14	26‐Jul	22‐Jul‐92	23‐Jul‐91
LV9	M	802	20.8	30.0	12	27‐Jul	02‐Jul‐92	03‐Jun‐91
LV12	M	818	18.8	26.0	10 or 13	03‐Aug	19‐Jun‐92	20‐Jun‐91
LV1	F	595	14.2	19.3	9	05‐Jul	05‐May‐92	26‐Mar‐91
LV7	F	719	15.7	26.8	No dip	24‐Jul	na	na
LV10	F(p)	882	14.8	27.5	10	31‐Jul	24‐Jun‐92	25‐Jun‐91
LV15	F	595	14.4	20.0	12	06‐Aug	21‐May‐92	22‐May‐91
S1	F	718	15.1	22.5	9	08‐Jul	na	18‐Apr‐91
S4	F	746	19.8	30.0	10	10‐Jul	03‐Jun‐92	04‐Jun‐91
S7	F	764	19.7	26.0	9	18‐Jul	27‐Apr‐92	28‐Apr‐91
S9	F	742	18.1	28.0	10	20‐Jul	01‐Jul‐92	02‐Jul‐91
S10	F(p)	752	21.3	32.0	12	21‐Jul	29‐Jun‐92	30‐Apr‐91
S11	F(p)	782	18.0	28.0	9	22‐Jul	26‐Mar‐92	27‐Mar‐91

Note

An ‘na’ is indicated where the minimum could not be distinguished.

### 2.2 Baleen plate collection and sample preparation

Fresh baleen plates were collected, and the longest plate from each whale was stored at −20°C until analysis.

All samples were pre‐treated using standard procedures for animal keratin samples (O'Connell & Hedges, 1999). Coarse materials were removed with a scalpel. The plates then received 30‐min ultrasonic bath treatments in distilled water, a solution of 2:1 methanol:chloroform repeated twice, a solution of 1:2 methanol:chloroform repeated twice, and distilled water repeated three times. Samples were air dried overnight and then freeze dried for several hours.

The length of the baleen plates, excluding the bristles, ranged from 14.2 cm to 21.3 cm depending on the size of the whale (Table 1). Two females were just under 600 cm in length. If these (immature) females are excluded, the average length of baleen plates across whales greater than 700 cm in length is 18.2 cm. Sampling of baleen plates was done at 1‐cm increments along the labial edge of the plate for the full length of the plate. Samples were standardized to weigh between 2 and 3 mg. Each sample was wrapped in a cleaned tin capsule (6 × 4 mm pressed tin capsules, standard weight, Elemental Microanalysis Ltd, UK) and stored in individual Eppendorf tubes labelled with the sample's identity.

### 2.3 Fish and zooplankton sample preparation

Whole cod, herring, and krill obtained from the Barents Sea in 2014 were homogenized, and 500ml of their tissue homogenate was stored at −20°C in separate Nalgene bottles. For sample analyses, ~1 cm^3^ of the sample was transferred into four 10 ml tubes, frozen at −80°C for 24 h, and freeze dried. After freeze drying, two sample replicates were weighed out at 1 mg, and two sample replicates were weighed out at 3 mg and wrapped in cleaned tin capsules (6 × 4 mm, pressed tin capsules, standard weight, Elemental Microanalysis Ltd, UK). The procedure of sample preparation was repeated a second time to obtain another complete set of four replicates for isotopic analysis.

### 2.4 Isotopic analysis

Isotopic analysis was performed at the Godwin Laboratory for Paleoclimate Research at the University of Cambridge, UK, using an automated carbon and nitrogen analyser coupled in continuous flow to an isotope‐ratio‐monitoring mass spectrometer (cf‐irm‐ms) (Costech 4010 elemental analyser connected to a Thermo Delta V mass spectrometer). Results are reported using the delta scale in units of ‘per mil’ (‰) relative to internationally accepted standards, VPDB for carbon and AIR for nitrogen.

### 2.5 Data treatment

Baleen growth was approximated as per methods of Mitani et al. (2006) for Pacific minke whales; the distance between isotope peaks was assumed to represent annual growth rate (Table 1). The annual growth rate was used, alongside with the date of capture to correspond with the root of the baleen plate (where attachment to the jaw occurs), to assign an estimated date to each isotope value measured along the length of baleen. The estimated dates assigned to the isotope measurements (which were taken 1 cm apart) were approximately one month apart; this is in line with the monthly growth rates in the literature (e.g. ~9.9 mm for male Pacific minke whales in Mitani et al., 2006 and ~9.2 mm in a female North Atlantic minke whale Riekenberg et al., 2020). These estimated dates were categorised into seasons based on boreal equinox and solstice dates. The season date ranges were 21 December to 20 March (Winter); 21 March to 20 June (Spring); 21 June to 20 September (Summer); and 21 September to 20 December (Autumn).

Statistical analysis was done with R 3.6.1 (R Core Team, 2019) and figures were produced using the package ‘lattice’ (Sarkar, 2008). Linear mixed‐effect (LME) models were chosen as the most appropriate analysis given the limits in sample size. The ‘lme’ function from the ‘nlme’ package (Pinheiro et al., 2019) was used to examine the association between either of the δ^13^C or δ^15^N isotope measurements along the length of baleen and season, sex, and site. Models were set up with season, sex and site as fixed effects, and whale ID as a random effect. All isotope values of either δ^13^C or δ^15^N, measured along the baleen plates, were used for LME models. The association between sex and isotope signatures could not be verified from the Svalbard location given the lack of samples from male whales at this site. The ‘corSpher’ correlation term was included in LMEs to account for autocorrelation in data points. This ‘corStruct’ class was chosen by testing whether addition of the correlation term improved the model fit, assessed via Aikake Information Criterion, against the basic model without a correlation structure.

Isotope values (δ^13^C and δ^15^N), from other North Atlantic minke whales and from their fish and zooplankton prey, were obtained from the literature (Table S1 and associated references). For the δ^13^C and δ^15^N isotope values reported in each study as well as those from the present study, the mean, standard deviation, and sample size were obtained and a composite mean and standard deviation was calculated as per Burton (2016). In studies where δ^13^C and δ^15^N isotope values were presented in a figure, the isotope values were obtained from the figure using ImageJ 1.53e (Rasband, 1997–2018).

Data supporting this publication are available at Eerkes‐Medrano et al. (2021).

## 3 RESULTS

**FIGURE 3:**
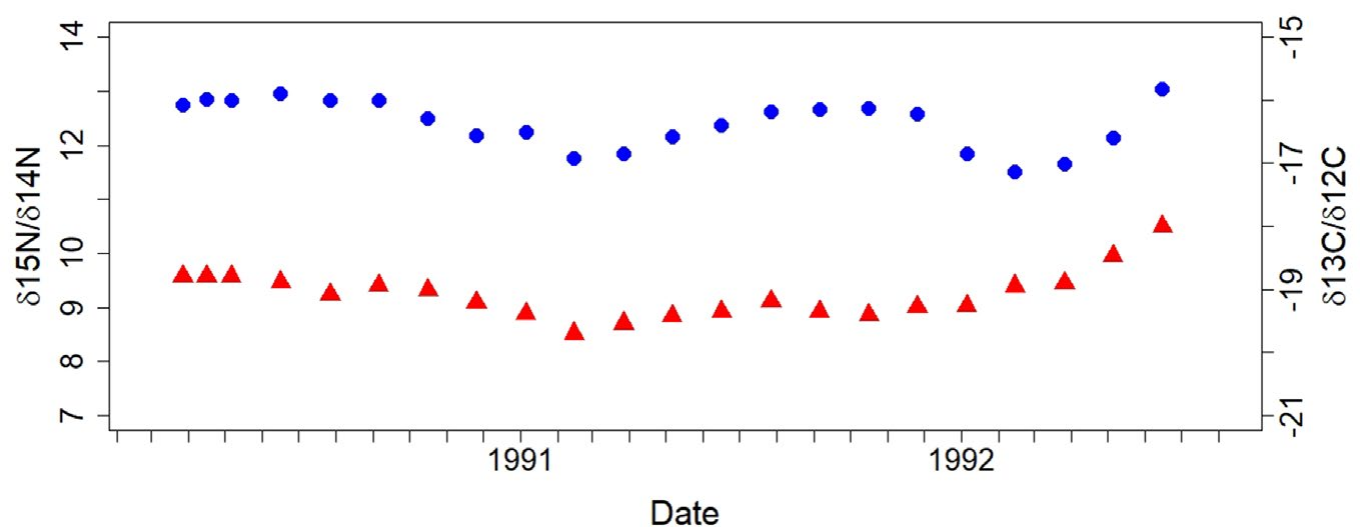
Examples of the variation in δ^15^N and δ^13^C isotope values along the length of baleen plates from one Svalbard female (S11). Blue dots are δ^15^N isotopes, and red triangles δ^13^C isotopes. Refer to the Appendix S3–S5 for isotope plots of the remaining individuals

**FIGURE 4:**
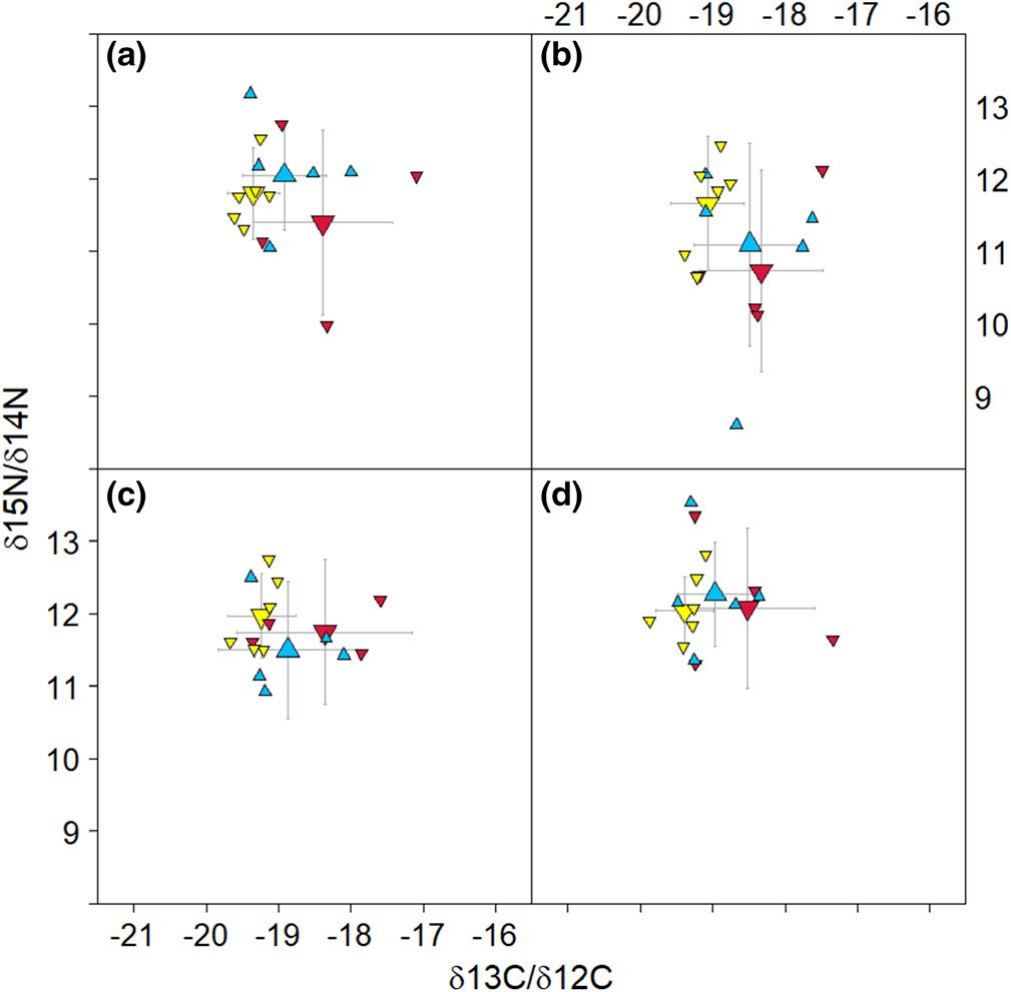
Plot of δ^15^N versus δ^13^C isotope values in baleen of minke whales over four seasons (a) winter, (b) spring, (c) summer, (d) autumn. The small triangles show mean isotope values for each whale, and the large triangles show grand mean and standard deviations for each group of whales. The seasons are defined following the temperate equinox and solstice dates (winter from 21 Dec to 20 March, spring from 21 March to 20 June, summer from 21 June to 20 September, and from autumn 21 September to 20 December). Symbol codes: Lofoten/Vesterålen females = red triangles, Lofoten/Vesterålen males = blue triangles, Svalbard females = yellow triangles

The average growth rate of baleen across all whales (>700 cm) is 11.0 ± 1.7 cm/year; broken down by sex, it is 12.8 ± 0.9 cm/year and 10.1 ± 1.2 cm/year for males and females, respectively. When plotted by estimated dates, nitrogen isotopes of most whales have a dip occurring in the spring (Table 1, Figure 3, and see Figures [Link], [Link], [Link] for isotopes plotted by distance in cm from gum). Differences in the spread of nitrogen isotope values between the groups of whales, and in particular between the estimated seasons, suggests differences between the Lofoten/Vesterålen (L/V) and Svalbard whales (Figures 4 and S2). The upper spread of δ^15^N values throughout the year (reaching between 13 and 14‰) is similar in all groups. At the lower spread of δ^15^N values there is greater variation, with lowest values occurring in spring and these being most pronounced in L/V whales (reaching between 7 and 8‰). L/V whales have the largest variation in nitrogen values, most notable from winter through summer, but evident regardless of the time of year. Svalbard females show the tightest range in nitrogen isotope values in all seasons, except for δ^15^N in L/V males in the autumn. The site and seasonal differences were supported in LME models by weak associations between δ^15^N isotope values and site (*β* = 0.72, CI = −0.10 to 1.54, *p* = .08) and between δ^15^N isotope values and season (*β* = 0.06, CI = −0.00 to 0.13, *p* = .06).

Carbon isotopes also suggest differences between whales from the two locations, with an association between δ^13^C values and the site (β = −0.86, CI = −1.64 to −0.08, *p*<0.05). The spread of δ^13^C values in scatterplots also seem to differ by season (Figures 4 and S1) but this association between δ^13^C and season was not picked up in LME models. With regards to patterns between whale groups, L/V females had the largest variation in δ^13^C values and Svalbard females had a spread of δ^13^C that was consistently narrow.

**FIGURE 5:**
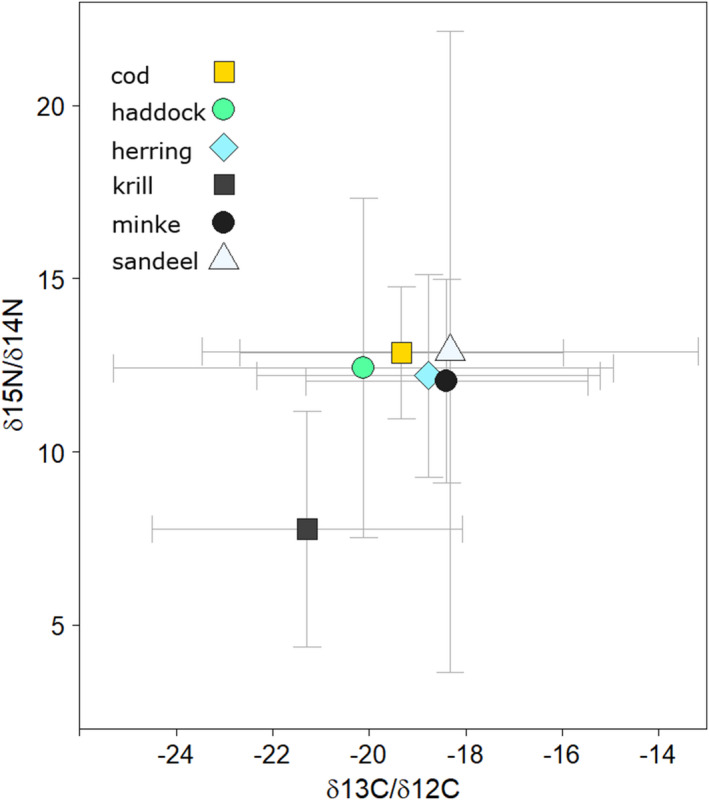
Composite mean and standard deviation for δ^15^N and δ^13^C isotope values for minke whales and potential prey items (cod, haddock, herring, sandeel, and krill) from the present study and studies in the literature. All literature values are for studies conducted in the North Atlantic (see references in the Appendix S1). The sample sizes for isotope values represented from each type of animal, including those measured in the present study, are minke = 2, cod = 5, herring = 7, haddock = 5, sandeel = 4, krill = 5

With regards to the isotope values obtained from the literature for minke whales and their prey, the composite mean values for fish and minke whales cluster within a range of 12 and 13 ‰ δ^15^N while krill are found at 7.7 ‰ (Figure 5).

## 4 DISCUSSION

The average rate of baleen growth from males in the present study was 12.8 ± 0.9 cm per year, in line with the growth rate of 12.9 ± 2.1 cm reported for male Pacific minke whales (Mitani et al., 2006) and the growth rate of ~11.9 cm reported for a single female North Atlantic minke whale (Riekenberg et al., 2020). The benefit in applying stable isotopic analysis to samples taken along the length of baleen plates is that a longer temporal record is obtained, as opposed to direct observations of feeding, samples from gut contents of dead animals, or faeces, which provide point samples of feeding activity. With an average length of 27.6 cm (in whales >700cm) from the base of the gum to the end of the bristles, the baleen plates represent 2.2 and 2.7 years of growth in male and female minke whales, respectively. If the free bristles are excluded from the baleen length measurement, then the average length of baleen plates is 18.2cm, representing 1.5 and 1.8 years of growth for males and females and in line with the 1.4‐year growth record estimated for baleen of male Pacific minke whales (Mitani et al., 2006) or assumed by Hobson et al. (2004) for North Atlantic minke whales.

The minke whale is a generalist and feeds on what is available (Skern‐Mauritzen et al., 2011). As an example, a study of 56 individuals taken from coastal areas of northern Norway and Russia shows that minke whales were flexible in their diet and adapted to local prey abundances, which included plankton, 0‐group fish (mainly herring (*Clupea harengus*), capelin (*Mallotus villosus*), cod (*Gadus morhua*), and haddock (*Melanogrammus aeglefinus*), pelagic fish (sandeel and saithe) and various demersal fish species (Skaug et al., 1997). In the latter study, 18 individuals taken from the coast of Lofoten/Vesterålen during July/August 1992 had 0‐group fish, herring and pelagic fish forming the largest‐by‐biomass diet items. The δ^15^N values overlap sufficiently amongst these types of fish (Figure 5) that the present study only distinguishes between periods when the North Atlantic minke whales eat fish or zooplankton, rather than attempting species identification of prey in the baleen records.

**FIGURE 6:**
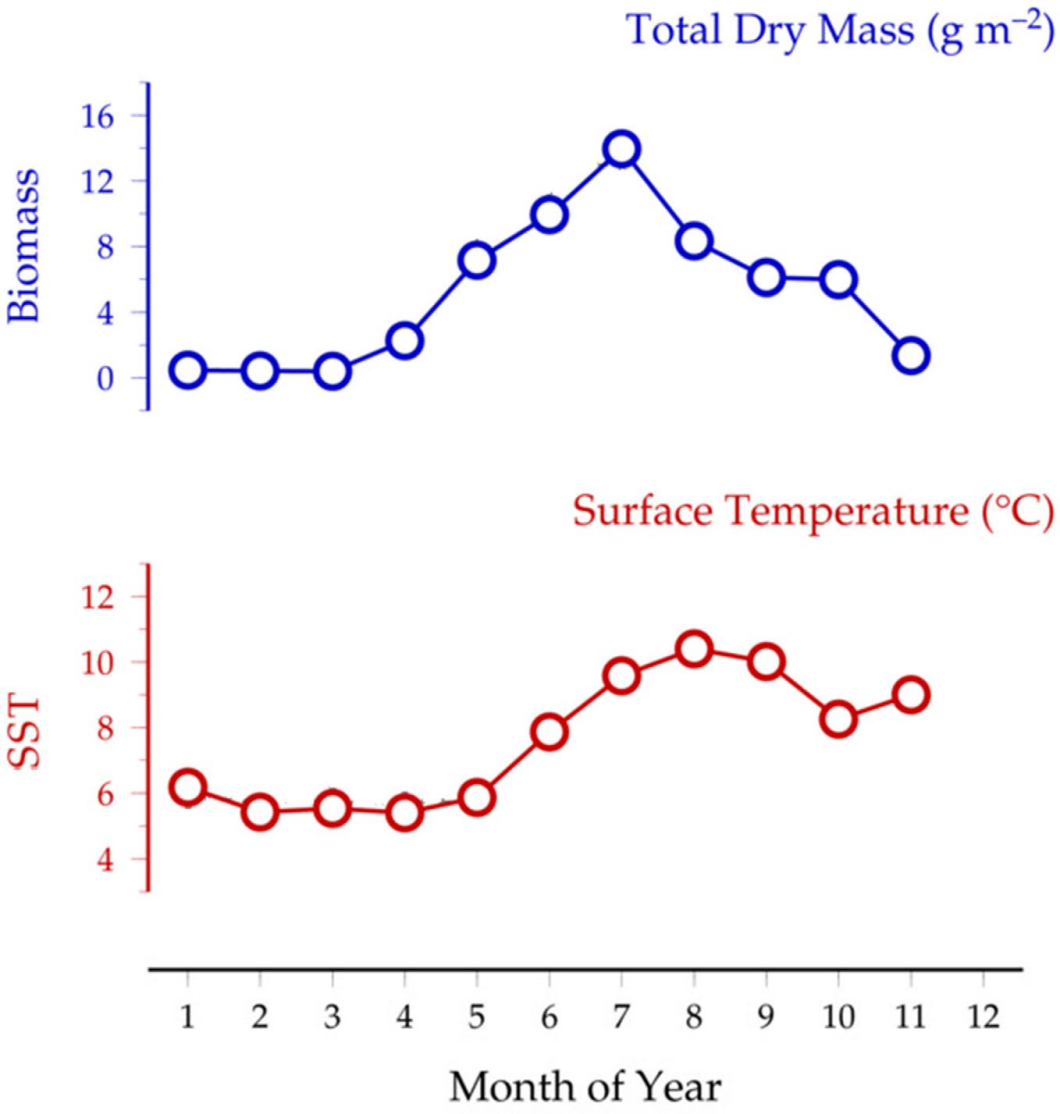
Seasonal cycle of zooplankton dry mass (blue) and sea surface temperature (red) from the Fugløya‐Bjørnøya Transect (70°30″N; 20°00″E–74°15″N; 19°10″E) (Adaptation of figure as featured in the METABASE Explorer https://www.st.nmfs.noaa.gov/copepod/time‐series/no‐50202/)

In line with observations for the Pacific minke whale (Mitani et al., 2006) and the single female North Atlantic minke whale (Riekenberg et al., 2020), the baleen plates for North Atlantic minke whales in the present study contain distinct δ^15^N depletions. For Lofoten/Vesterålen individuals, the timing of δ^15^N depletions occurs predominantly between May to July (Table 1), which can be interpreted to reflect annual shifts in the whale's foraging that coincide with the annual zooplankton productivity peak. Timing of this zooplankton productivity peak can be confirmed by observations from the Fugløya‐Bjørnøya long‐term zooplankton time series, located a couple of degrees north of Lofoten; here, the annual increase in zooplankton biomass starts in April and peaks in July (Figure 6). Given the findings from Skaug et al. (1997) that minke whales taken from Lofoten/Vesterålen in 1992 contained mainly herring and pelagics in their stomachs, and, given that spring‐spawning herring are known to track zooplankton dynamics in the Norwegian sea (Broms et al., 2012), it is likely the elevated periods in δ^15^N reflect a switch to forage fish species such as herring. While Svalbard individuals also exhibit δ^15^N depletions, the δ^15^N do not drop so much as from Lofoten/Vesterålen whales. An overall tighter distribution of δ^15^N values for Svalbard whales suggests that Svalbard whales may have a more consistent diet than Lofoten/Vesterålen whales.

Baleen whales are thought to eat much less or not at all during the winter breeding season. In terrestrial vertebrates it is widely accepted that fasting periods lead to elevated δ^15^N in tissues (Hobson et al., 1993; Polischuck et al., 2001), but the evidence for baleen whales is inconclusive. Best and Schell (1996) speculate that late winter/spring peaks in δ^15^N oscillations from southern right whales (*Eubalena australis*) are indicative of starvation through winter, but note that δ^15^N peaks in spring also arise due to feeding. In measurements from the baleen plates of fin and bowhead whales, Aguilar et al. (2014) and Matthews and Ferguson (2015) found increased δ^15^N during summer feeding and decreased δ^15^N values during the winter, thus contradicting fasting predictions. Based on δ^15^N and δ^13^C from skin samples of North Atlantic blue (*Balaenoptera musculus*), fin (*Balaenoptera physalus*) and sei (*Balaenoptera borealis*) whales, Silva et al. (2019) report that winter‐feeding appears common. Riekenberg et al. (2020) examined δ^15^N amino acids in minke, humpback, and fin whales and reported greater evidence that fin whales experience food stress than for either the humpback or minke whale. Mitani et al. (2006) reported no detectable impact of fasting in the isotopic record in Pacific minke whales. In the present study, the patterns of δ^15^N in baleen plates fail to suggest a detectable fasting period.

Trends in stable carbon isotopes have been used to study migrations in some baleen whales, such as southern right whales, whose baleen plates can be >200 cm in length and who conduct their seasonal migrations in the southern hemisphere, where strong gradients in plankton δ^13^C values can be detected. In these whales marked, δ^13^C oscillations (ranging up to 8 ‰) in baleen plates of four adults were suggested to reflect the whales’ annual north‐south migrations (Best & Schell, 1996). In bowhead whales, which also have long baleen plates (up to 400cm in adult whales), stable carbon isotopes have been used to track east–west migrations between winter areas in the western Bering Sea (>180°W) and summer areas in the eastern Beaufort Sea (136°W) (Schell et al., 1989). For these whales, δ^13^C oscillations ranged up to 4 ‰. In Pacific minke whales, the baleen plates are a fraction of the size (~20cm), for which Mitani et al. (2006) stated that mature whales from their investigations lacked the strong oscillations in δ^13^C that have been reported for southern right whales. They suggested the lack of marked δ^13^C oscillations in baleen plates of Pacific minke whales is because the gradient seen from lower latitudes to the poles in plankton δ^13^C values, is only 0.1 times in the northern hemisphere compared to the southern hemisphere. In addition to the potential use of δ^13^C to reflect latitudinal gradients in plankton, δ^13^C values are also reported to vary amongst prey items from coastal habitats versus offshore habitats, e.g. enriched δ^13^C values across trophic levels have been reported from animals sampled closer to shore (Miller et al., 2008; Sydeman et al., 1997). Long‐finned pilot whales (*Globicephala melas*) feeding on coastal prey reflect different δ^13^C values than those feeding in deeper habitats (Monteiro et al., 2015). In addition to the above, coastal upwelling (Miller et al., 2008) and season (Sadler et al., 2012) are other factors associated with changes in δ^13^C values.

In the present study of North Atlantic minke whales, the greatest range in δ^13^C occurred in spring and summer. The δ^13^C signatures were remarkably similar throughout the year with little variation for Svalbard females (Figures 4 and S1, S5). The Lofoten/Vesterålen whales show greater variation throughout the year, and particularly for the females, with a larger spread in δ^13^C values in spring (March–June) and summer (June–September). The Lofoten/Vesterålen males, on the other hand, show somewhat less variance than the females, except for in spring (March–June) (Figures 4 and S1, S3, S4). Although the graphical examination appears to show visible seasonal patterns in δ^13^C values, these were not supported statistically and may require greater sample sizes to be resolved. There was a significant association between δ^13^C and site, which could reflect differences in foraging locations between whales of different sites. For example, the Lofoten/Vesterålen animals may shift between on‐shelf and off‐shelf foraging, whereas Svalbard animals may be more consistent in their foraging locations. The seasonal patterns of δ^13^C observed in plots (Figures 4 and [Link], [Link], [Link], [Link]) could suggest migratory activity (as per Best & Schell, 1996), or that enriched δ^13^C values from spring and summer, are a result of different plankton species in the water column (as per Sadler et al., 2012). Indeed, other marine studies report the primary productivity peak occurring in summer as the main reason for δ^13^C enrichment (Sadler et al., 2012). However, without a significant association between δ^13^C and seasons, there is insufficient evidence to verify either speculation.

The stable isotopic signatures of whale baleen can be useful to reveal patterns in year‐round foraging habits. However, even with a greater sample size, the baleen record of Northeast Atlantic minke whales may be unsuitable to provide accurate insights into their migrations. The field of dietary studies continues to evolve, with new techniques that provide greater accuracy in estimating trophic position and metabolic condition (e.g. amino acid nitrogen isotopic analysis, O’Connell, 2017; Riekenberg et al., 2020) and an increasing use of inter‐disciplinary approaches that provide greater insights in dietary studies (e.g. biochemical and molecular approaches, Horswill et al., 2018). As this research is undertaken, it can benefit from questions and hypotheses formulated on the natural history of minke whales.

The paradigm of mysticete seasonal migrations between high‐latitude summer feeding grounds and low‐latitude wintertime breeding grounds is subject to an on‐going debate that questions whether all individuals within a population migrate (Corkeron & Connor, 1999; Geijer et al., 2016; Kavanagh et al., 2018). Examining the case for the Northeast Atlantic minke whale, an implication that all whales undertake a wintertime migration and, in so doing, leave the abundant food resources in the Norwegian and Barents Seas, presents the question of why males and, particularly, immature animals of both sexes might migrate. Folkow and Blix (1992) show that young minke whales are thermoneutral in water close to freezing, which suggests that migration into warm water is to no energetic advantage. In Norwegian waters there is no direct information on to the newborn minke calf, which measures 2.74 m at birth (Jonsgård, 1951), but newborn narwhal (*Monodon monoceros*) and beluga whale (*Delphinapterus leucas*), which measure 1.6 m and 1.5 m, respectively, thrive and grow in ice‐water. Killer whale (*Orcinus orca*) calves have been observed on the Norwegian coast at 63°N in January (Christensen, 1978).

For the sake of the argument, if minke whales are not migrating to the tropics *en masse*, why are they not caught or seen, in the Norwegian Sea in winter? This may be due to the darkness; ship‐based activities, such as winter fisheries for cod (*Gadus morhua*) and herring (*Clupea harengus*) occurring near the coast; rough sea conditions; and most importantly, the fact that the minke whales in the northern hemisphere do not spout when they surface to breathe. All this make them extremely difficult to spot, even when on lookout under optimal (dead calm) sea‐state conditions during summer.

Conversely, what observations are reported at lower latitudes? There is a report of 200–250 minke whales sighted between 20 and 30°N and 30 and 40°W in May (Slijper et al., 1964). However, at those coordinates, neither southern nor northern hemisphere minke whales would be expected to be found in May when the whales should be feeding at higher latitudes. These sightings were made by laymen; thus it is possible the animals were not minke whales. Visser et al. (2011), who performed 366 surveys off the Azores during the spring bloom period (March‐June), only observed six minke whales out of a total of 107 baleen whales. Krill becomes suitable for whale consumption in the Azores in April/May (Visser et al., 2011), whereas the larger baleen whales seem to congregate and linger in the area to take advantage of the krill resources (Silva et al., 2013), and the minke whales, which are not dependent on krill, are not abundant in the area (Visser et al., 2011).

As for efforts using biotelemetry and/or acoustic surveys, Vikingsson and Heide‐Jørgensen (2015) satellite‐tagged three minke whales of unknown sex and age off Iceland in August and September. All three travelled south. Contact was lost with two of them in early October and November in the mid‐Atlantic off the coast of England, whereas one was tracked to where Slijper et al. (1964) reported the appearance of a large group of whales in the mid‐Atlantic at about 28°N in early December. Heide‐Jørgensen et al. (2001) tagged two whales (one in September 1994 and one in August 1999) with satellite‐linked transmitters outside Lofoten. One large whale remained in the area until contact was lost on 5 October 1994 while the other, a small whale, operated along the coast of northern Norway until contact was lost on 8 September 1999. Risch et al. (2014) obtained some indirect (acoustic) evidence for the presence of minke whales at the mid‐Atlantic ridge southwest of the Azores in December–February. However, Folkow and Blix (1991) conducted a sighting and acoustic survey from Oslo to Montevideo in December and from Rio de Janeiro to Las Palmas in March. In spite of having dropped 49 hydrophone buoys and made 240 h of observations along the track line in the mid‐Atlantic, only two observations of a minke whale were made and sound pulses similar to those previously described for the minke whale (e.g. Winn & Perkins, 1976) were detected only once. Finally, some minke whales were caught outside Lofoten, despite detrimental conditions in November, in the period from 1938 to 1949, when minke whales were harvested commercially throughout the year (Jonsgård, 1951).

In light of the evidence concerning minke whale biology and movements, an alternative hypothesis suggests that minke whales are not migrating to the tropics *en masse*. Males and immature animals may remain for most of the year in Norwegian waters. A similar view, suggesting that not all individuals migrate, has been presented by others (Corkeron & Connor, 1999; Jonsgård, 1966; Kavanagh et al., 2018), but has received little attention. One of these studies in particular is based on two wintertime sightings (once in November and once in March) of minke whale calves in deep waters off the shelf edge from Ireland at 53.684°N and 55.523°N. The individuals were approximately 2.5 m and 3.5 m and in company of an adult, so thought to be unweaned calves (Kavanagh et al., 2018). Potentially it is mainly the pregnant females ready to give birth that travel to lower latitudes, not of a requirement for warmer water, but far enough to avoid predation from killer whales as suggested by Corkeron and Connor (1999). These predators are abundant in the Norwegian Sea where they feed on herring and mackerel (*Scomber scombrus*) during summer and autumn (Nøttestad et al., 2014) and along the west coast of Norway in winter (Øien, 1988).

Age determination in northern minke whales is uncertain (Christensen, 1995), and therefore also the age at sexual maturity. Kato (1987) found that 50% of southern female minke whales became sexually mature at the age of 6 years, whereas 100% were mature at the age of 10 years. The same appears true for the whales of the northeast Atlantic (Olsen & Sunde, 2002). This would then mean that immature animals comprise a great number of animals that may have no need to be in the tropics in winter. It follows, that even if only these animals remain in the Norwegian Sea throughout the year, it will have a significant impact on the food webs of the area, which has hitherto not been accounted for. Further research applying advancements in methods, new technologies and interdisciplinary approaches might better support the alternative hypothesis to the seasonal migrations of minke whales by examining specifically whether the sexes and age classes of minke whales exhibit differences in movements.

## AUTHOR CONTRIBUTIONS

**Dafne Eerkes‐Medrano:** Conceptualization (equal); Data curation (lead); Formal analysis (lead); Methodology (lead); Writing‐original draft (lead); Writing‐review & editing (equal). **David C. Aldridge:** Conceptualization (equal); Data curation (supporting); Formal analysis (supporting); Methodology (supporting); Writing‐original draft (supporting); Writing‐review & editing (equal). **Arnoldus S. Blix:** Conceptualization (equal); Data curation (supporting); Formal analysis (supporting); Methodology (supporting); Writing‐original draft (supporting); Writing‐review & editing (equal).

### DATA AVAILABILITY STATEMENT

Data supporting this article are available via *Pangaea* at Eerkes‐Medrano et al. (2021).

## Supporting information

Figure S1

Figure S2

Figure S3

Figure S4

Figure S5

Supplementary Material
