# Supplementary figures and images for: North Atlantic minke whale (Balaenoptera acutorostrata) feeding habits and migrations evaluated by stable isotope analysis of baleen
Source: Ecol Evol. 2021 Oct 24;11(22):16344–53. doi: 10.1002/ece3.8224 (PMC8601907; doi:10.1002/ece3.8224)

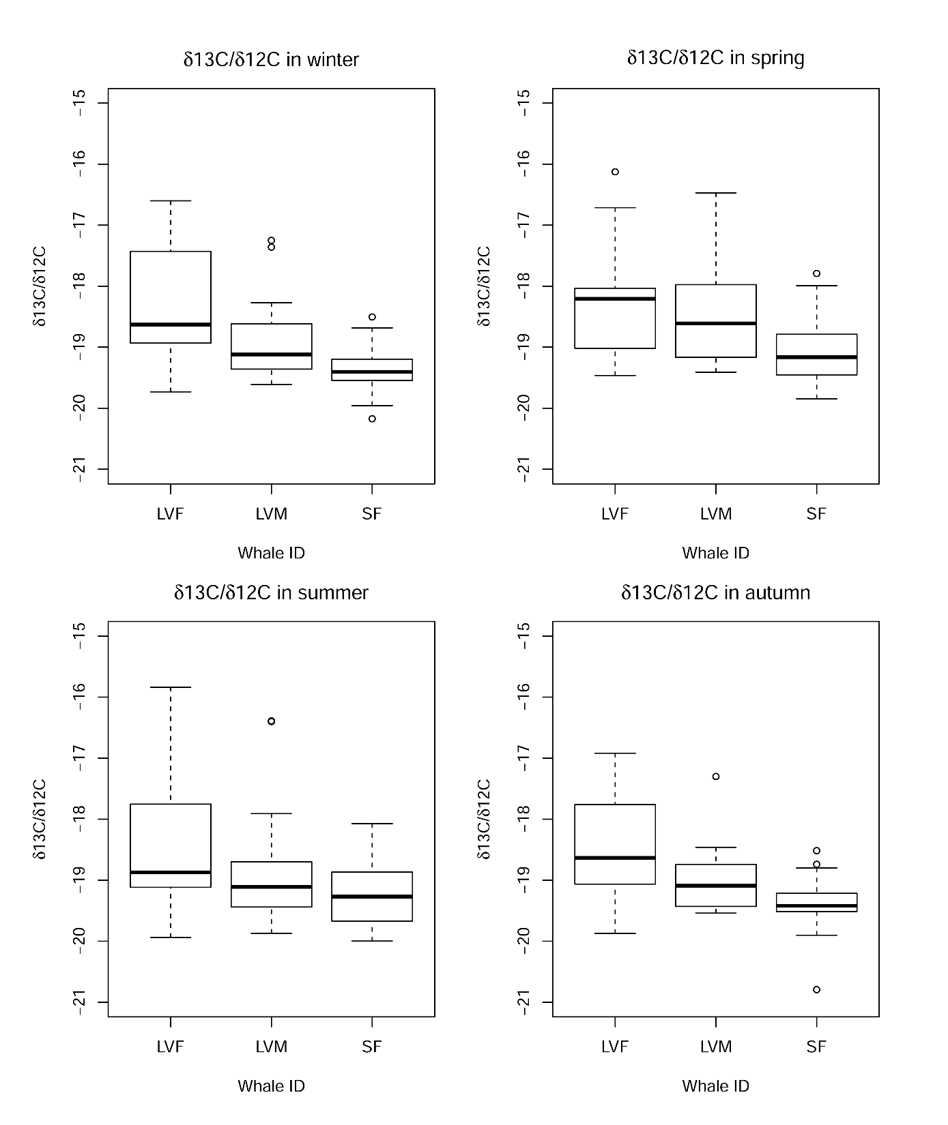

Supplement: Supplementary file 1 — Figure S1 [file ECE3-11-16344-s004.tif]

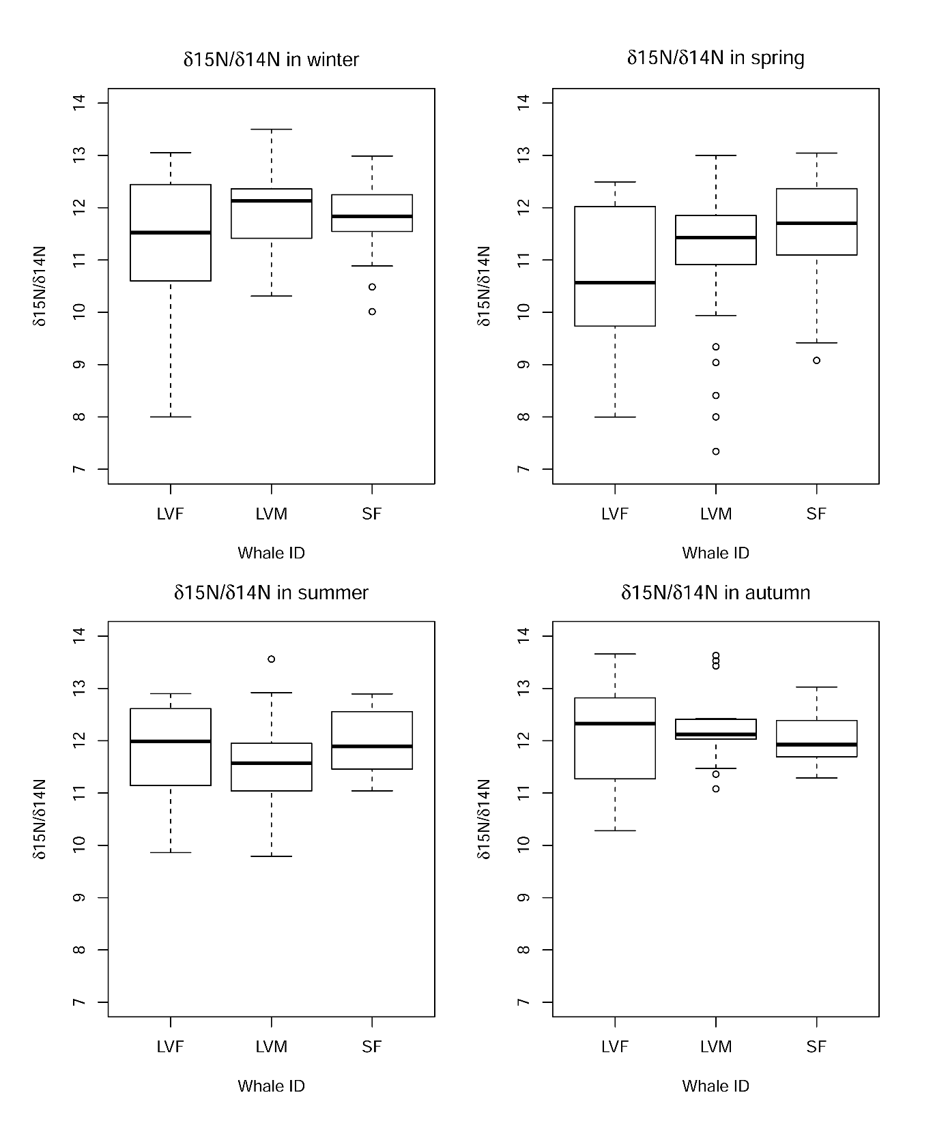

Supplement: Supplementary file 2 — Figure S2 [file ECE3-11-16344-s005.tif]

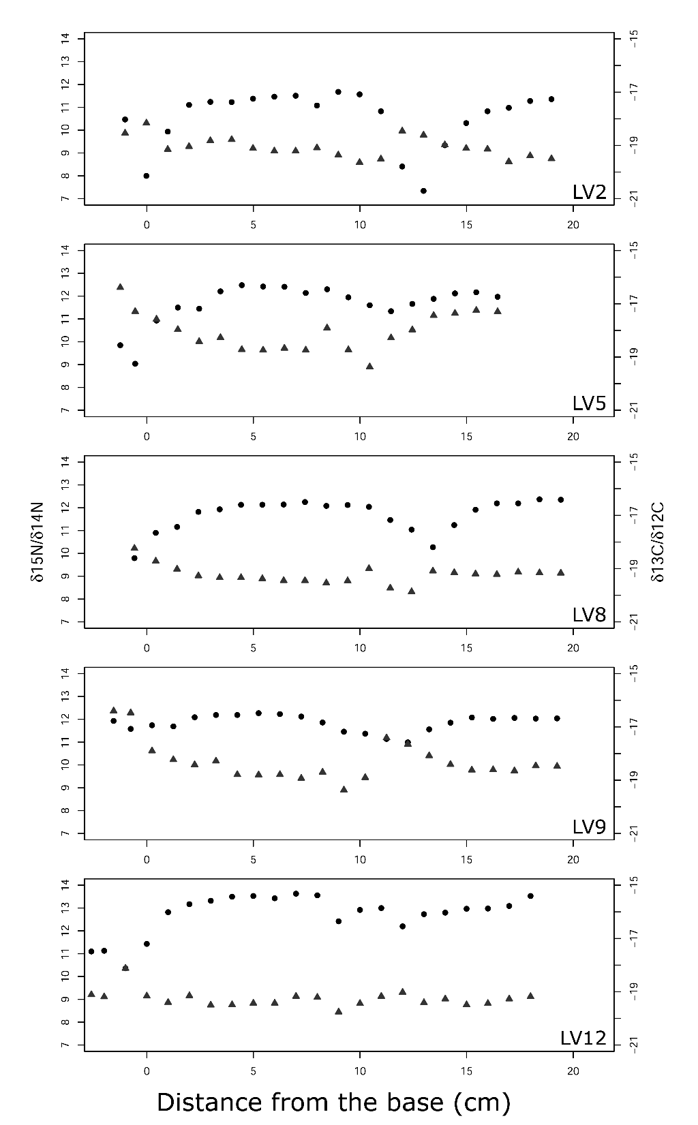

Supplement: Supplementary file 3 — Figure S3 [file ECE3-11-16344-s002.tif]

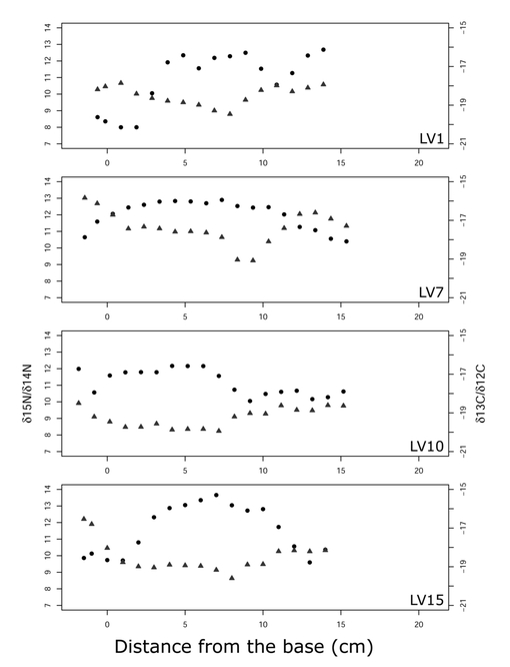

Supplement: Supplementary file 4 — Figure S4 [file ECE3-11-16344-s006.tif]

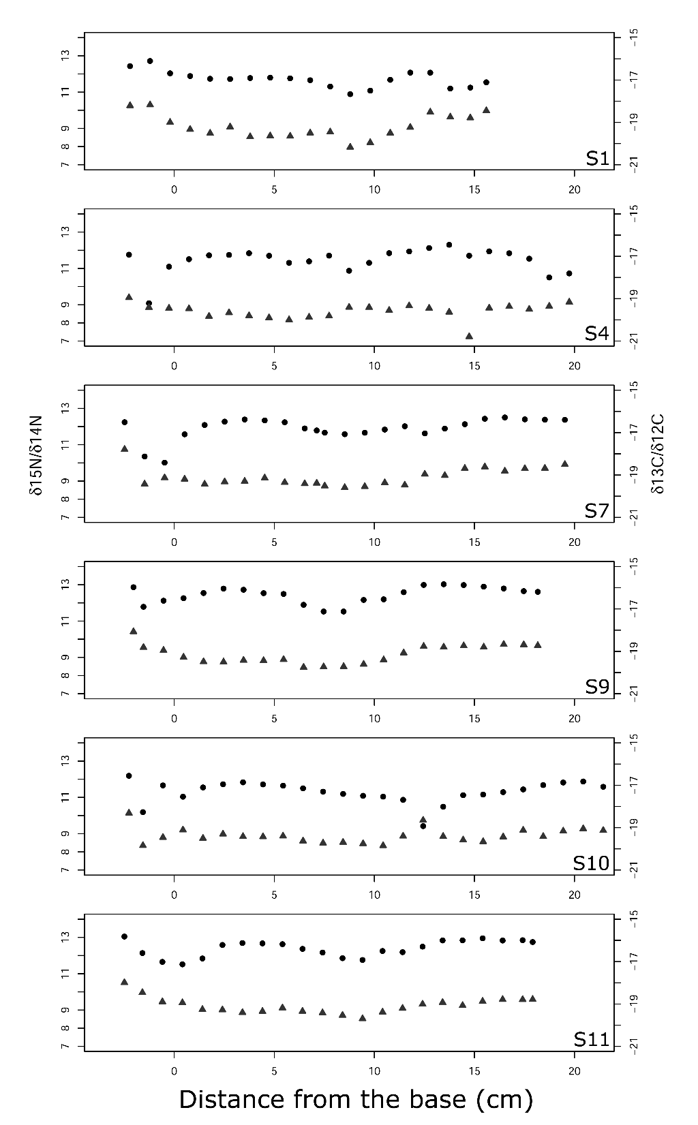

Supplement: Supplementary file 5 — Figure S5 [file ECE3-11-16344-s001.tif]
