# Supplementary material for: North Atlantic minke whale (Balaenoptera acutorostrata) feeding habits and migrations evaluated by stable isotope analysis of baleen
Source: Ecol Evol. 2021 Oct 24;11(22):16344–53. doi: 10.1002/ece3.8224 (PMC8601907; doi:10.1002/ece3.8224)
Supplement: Supplementary file 6 — Supplementary Material [file ECE3-11-16344-s003.docx]

**Appendix S1**


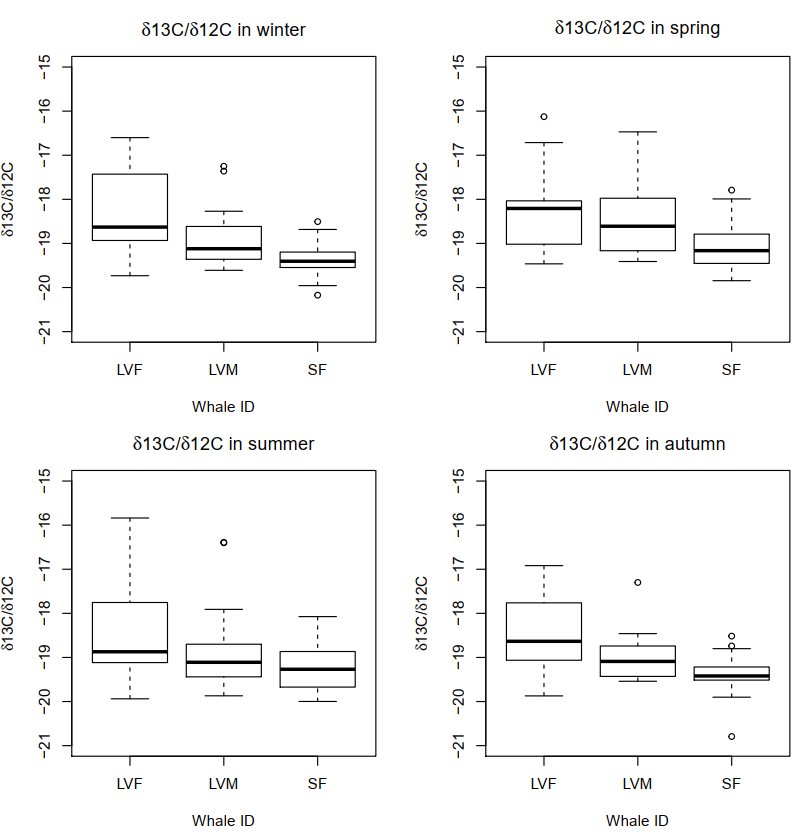


**Fig. S1.** δ^13^C isotope values in minke whale baleen in the four seasons. LVF = Lofoten/Vesterålen females; LVM = Lofoten/Vesterålen males; SF = Svalbard females. The seasons are defined following the temperate equinox and solstice dates (winter from 21 Dec to 20 March, spring from 21 March to 20 June, summer from 21 June to 20 September, autumn 21 September to 20 December).


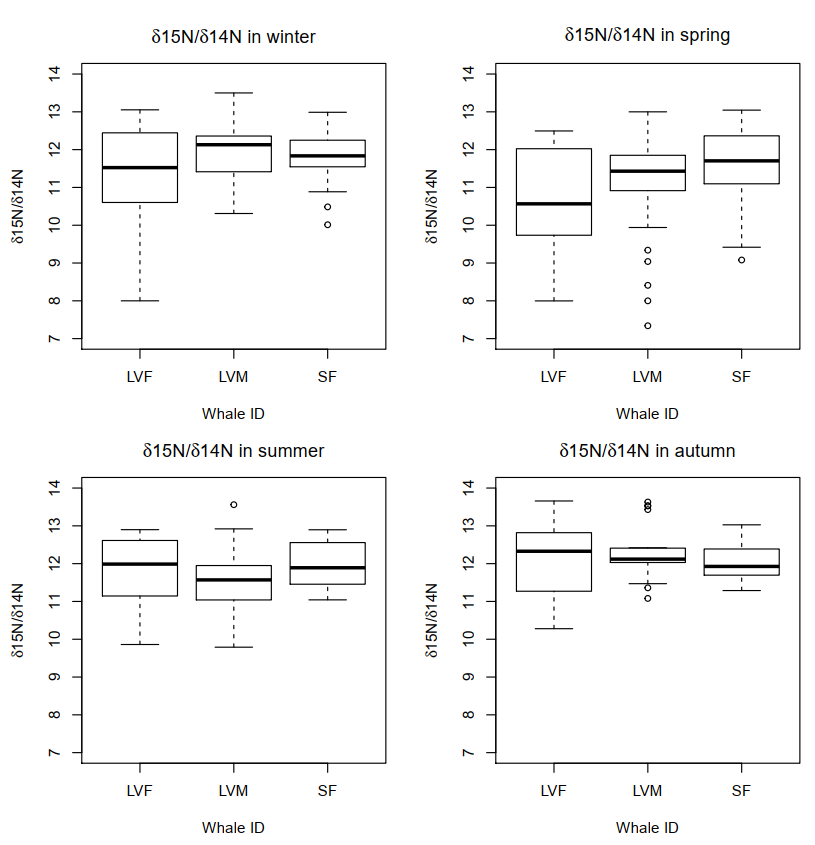


**Fig. S2.** δ^15^N isotope values in minke whale baleen in the four seasons. The seasons are defined following the temperate equinox and solstice dates (winter from 21 Dec to 20 March, spring from 21 March to 20 June, summer from 21 June to 20 September, autumn 21 September to 20 December). Symbols as for Fig. S1.


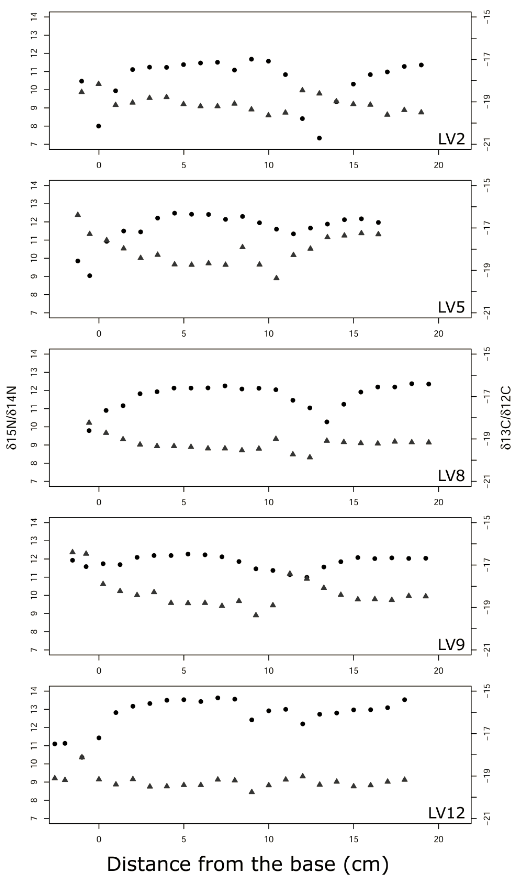


Figure S3. Variation in δ^15^N and δ^13^C values along the growth axis, measured in cm from the gum, from baleen plates of five Lofoten/Vesterålen males. Negative values indicate baleen growth in the soft rubbery layer of the Zwischensubstanz (Fudge et al. 2009), and positive values indicate the baleen growing beyond the Zwischensubstanz. The δ^15^N are represented by circles and δ^13^C by triangles.


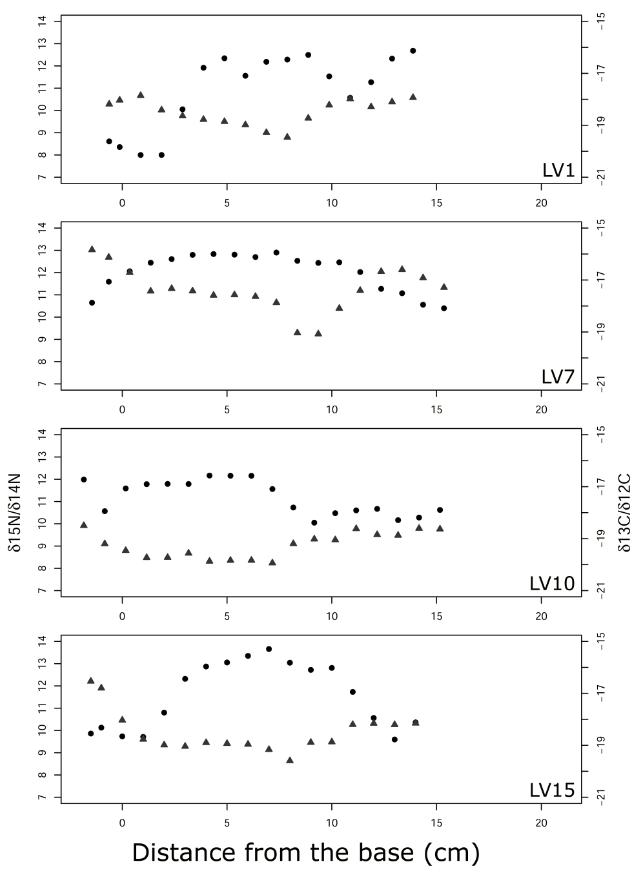


Figure S4. Variation in δ^15^N and δ^13^C values along the growth axis, measured in cm from the gum, from baleen plates of five Lofoten/Vesterålen females. Negative values indicate baleen growth in the soft rubbery layer of the Zwischensubstanz (Fudge et al. 2009), and positive values indicate the baleen growing beyond the Zwischensubstanz. The δ^15^N are represented by circles and δ^13^C by triangles.


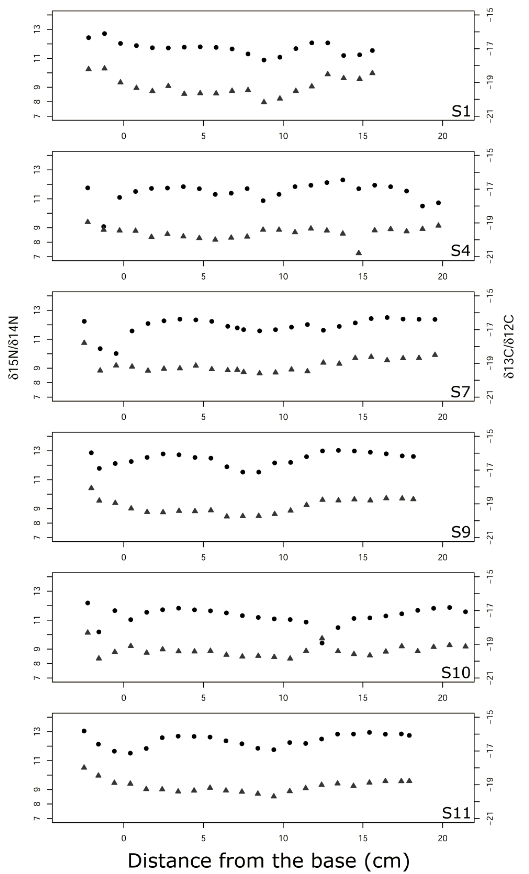


Figure S5. Variation in δ^15^N and δ^13^C values along the growth axis, measured in cm from the gum, from baleen plates of six Svalbard females. Negative values indicate baleen growth in the soft rubbery layer of the Zwischensubstanz (Fudge et al. 2009), and positive values indicate the baleen growing beyond the Zwischensubstanz. The δ^15^N are represented by circles and δ^13^C by triangles

**Studies used to make Figure 5**

**Table S1**. Literature used to create Figure 5. The separate studies providing information for each animal are cited

| **Animal** | **Studies used** |
| --- | --- |
| North Atlantic minke whale | Present study; Hobson et al. (2004); Riekenberg et al. (2020); Ryan et al. (2013) |
| Cod | Present study; Petursdottir et al. (2012); Ramsvatn & Pedersen (2012); Sarà et al. (2009) |
| Herring | Present study; Das et al. (2003); Kurle (2002); Caut et al. (2011); Ryan et al. (2014) |
| Haddock | Das et al. (2003); Petursdottir et al. (2012); Ramsvatn & Pedersen (2012); Sarà et al. (2009) |
| Sandeel | Das et al. (2003); Petursdottir et al. (2012); Sarà et al. (2009) |
| Krill | Present study; Petursdottir et al. (2012); Ryan et al. (2014) |

References for table A1

Caut S, Laran S, Garcia-Hartmann E, Das K (2011) Stable isotopes of captive cetaceans (killer whales and bottlenose dolphins). J Exp Biol 214(4):538-45.

Das K, Lepoint G, Leroy Y, Bouquegneau JM (2003) Marine mammals from the southern North Sea: feeding ecology data from δ13C and δ15N measurements. Mar Ecol Prog Ser 263:287-98.

Hobson KA, Riget FF, Outridge PM, Dietz R, Born E (2004) Baleen as a biomonitor of mercury content and dietary history of North Atlantic minke whales (*Balaenoptera acutorostrata*): combining elemental and stable isotope approaches. Sci Total Environ 331: 69-82.

Kurle CM (2002) Stable-isotope ratios of blood components from captive northern fur seals (*Callorhinus ursinus*) and their diet: applications for studying the foraging ecology of wild otariids. Can J Zool 80(5):902-9.

Petursdottir H, Falk-Petersen S, Gislason A (2012) Trophic interactions of meso- and macrozooplankton and fish in the Iceland Sea as evaluated by fatty acid and stable isotope analysis. ICES J Mar Sci 69(7):1277-88.

Ramsvatn S, Pedersen T (2012) Ontogenetic niche changes in haddock *Melanogrammus aeglefinus* reflected by stable isotope signatures, δ13C and δ15N. Mar Ecol Prog Ser 451:175-85.

Riekenberg PM, Camalich J, Svensson E, IJsseldijk LL, Brasseur SM, Witbaard R, Leopold MF, Rebolledo EB, Middelburg JJ, van der Meer M, Damste JS. (2020) Reconstructing the diet, trophic level, and migration pattern of Mysticete whales based on baleen isotopic composition. bioRxiv. doi: <https://doi.org/10.1101/2020.10.04.301341>.

Ryan C, McHugh B, Trueman CN, Sabin R, Deaville R, Harrod C, Berrow SD, Ian O (2013) Stable isotope analysis of baleen reveals resource partitioning among sympatric rorquals and population structure in fin whales. Mar Ecol Prog Ser 479:251-61.

Ryan C, Berrow SD, McHugh B, O'Donnell C, Trueman CN, O'Connor I (2014) Prey preferences of sympatric fin (*Balaenoptera physalus*) and humpback (*Megaptera novaeangliae*) whales revealed by stable isotope mixing models. Mar Mamm Sci 30(1):242-58.

Sarà G, De Pirro M, Sprovieri M, Rumolo P, Halldórsson HP, Svavarsson J (2009) Carbon and nitrogen stable isotopic inventory of the most abundant demersal fish captured by benthic gears in southwestern Iceland (North Atlantic). Helgol Mar Res 63(4):309-15.
